# Total Occlusion of Abdominal Aorta in Takayasu Arteritis

**DOI:** 10.7759/cureus.5058

**Published:** 2019-07-01

**Authors:** Rafiullah Jan, Salman Zahid, Syed M Owais, Fahad Khan, Zahid A Awan

**Affiliations:** 1 Cardiology, Hayatabad Medical Complex, Peshawar, PAK; 2 Internal Medicine, Hayatabad Medical Complex, Peshawar, PAK; 3 Internal Medicine, Khyber Teaching Hospital, Peshawar, PAK

**Keywords:** takayasu arteritis, abdominal aorta, claudication

## Abstract

Takayasu arteritis is a type of large vessel vasculitis that mainly affects the aorta and its major branches. The disease can have a myriad of manifestations ranging from non-specific symptoms of low-grade fever and weight loss to lower limb claudication. A 21-year-old woman presented with uncontrolled hypertension for the last six months. The CT aortogram revealed total occlusion of the abdominal aorta with collateral vessels formed by the right and left internal mammary artery. We present a case of Takayasu arteritis in a 21-year-old woman with complete obstruction of the abdominal aorta. She was treated only with oral medications. The associated review of the literature is also discussed.

## Introduction

Takayasu arteritis is a type of large vessel vasculitis that mainly affects the aorta and its major branches. In about 90% of cases, the disease affects women with an age of onset between 10-40 [1]. The first case of Takayasu was reported in 1908 by a Japanese Ophthalmologist Dr. Takayasu. The prevalence of this disease is highest in the Asian population. For instance, in Japan 150 new cases occur each year [2]. In contrast, only one to three cases per year per million population occurs in the US and Europe [3]. The disease can have a myriad of manifestations ranging from non-specific symptoms of low-grade fever and weight loss to lower limb claudication. Two distinct stages of the disease have been observed, the first one is a “pre-pulseless phase” in which there are non-specific inflammatory symptoms. This stage is followed by a more chronic phase which is characterized by symptoms due to vascular occlusions [4]. The hallmark feature of the disease is diminished or absent pulses which occur in 84-96% of cases. Other features include vascular bruits, hypertension, Takayasu retinopathy, aortic regurgitation, congestive cardiac failure, neurological features, dyspnea, headache, myocardial ischemia and erythema nodosum [5].

We present a case of Takayasu arteritis in a 21-year old lady with complete occlusion of the abdominal aorta. She was only treated with medications. The associated review of the literature is also discussed.

## Case presentation

**Figure 1:**
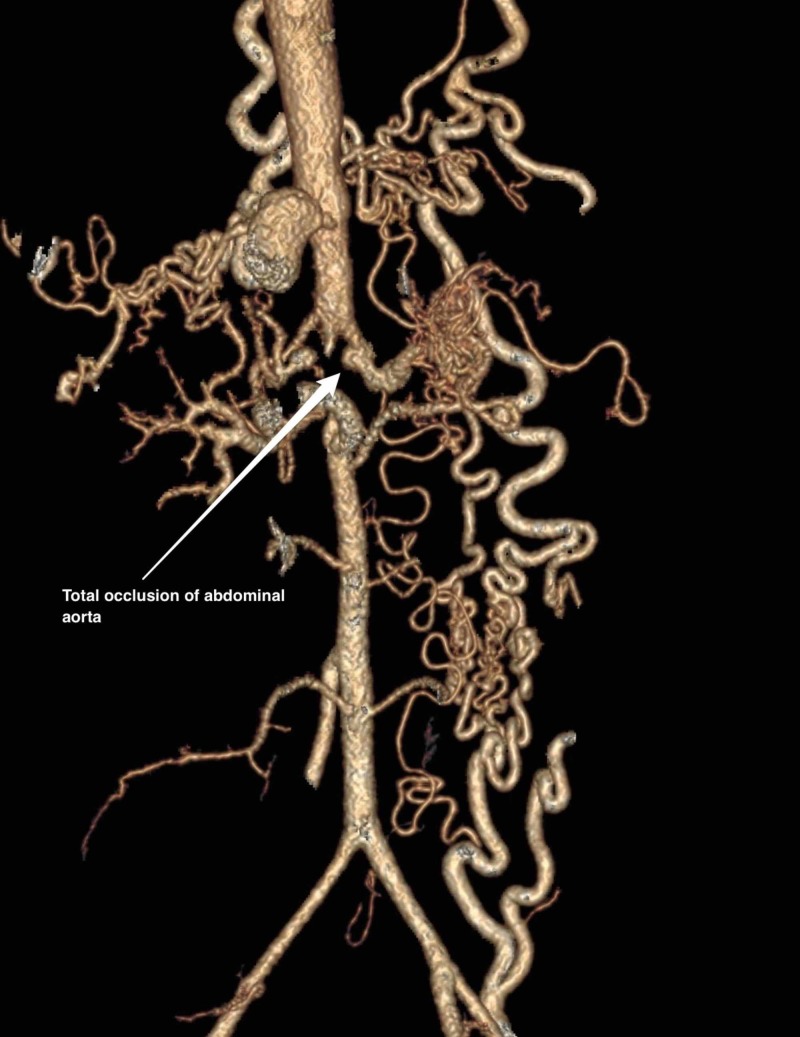
Computed tomography aortogram showing total occlusion of the abdominal aorta

**Figure 2:**
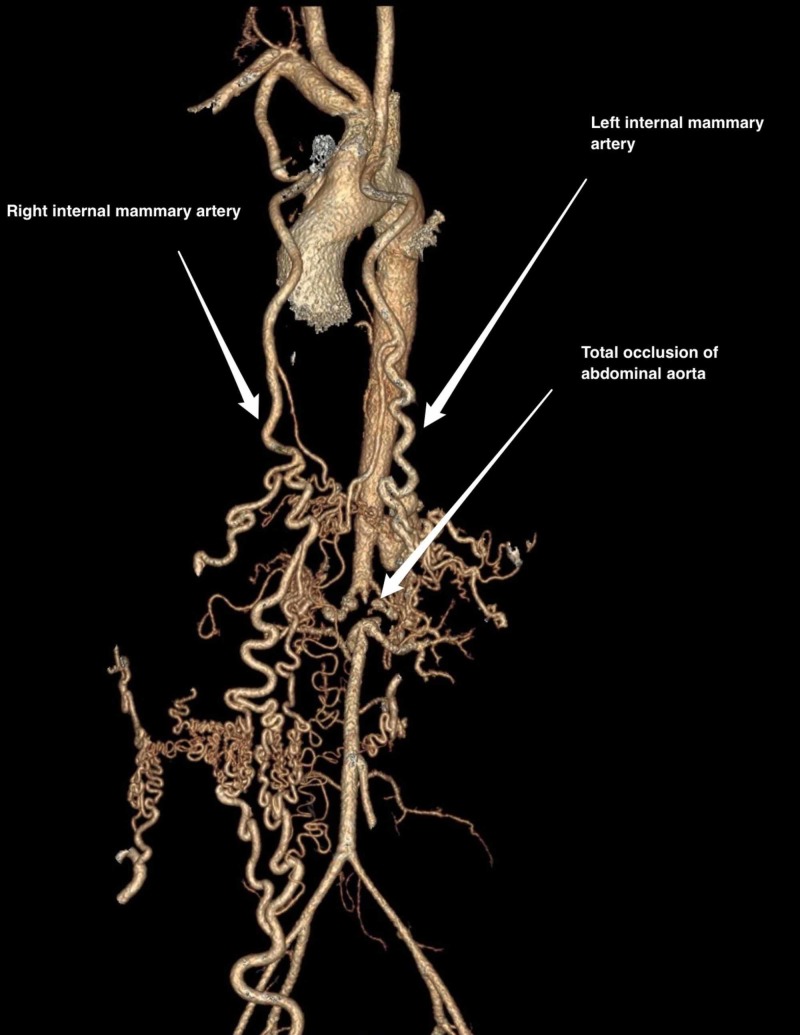
Computed tomography aortogram showing collateral vessels formed by the right and left internal mammary artery

A 21-year old Pakistani woman presented with uncontrolled hypertension for the last six months despite taking three anti-hypertensive drugs. She also complained of progressive lower limb claudication. On a review of systems, she had low-grade fever, fatigue, and unintentional weight loss. On physical examination, blood pressure in both upper extremities was 220/110. Femoral pulses were absent bilaterally. Laboratory investigations were unremarkable except for a raised erythrocyte sedimentation rate (ESR; 55mm) and elevated C-reactive protein (CRP; 20mg/dl, n<5mg/dl). To exclude coarctation of aorta, Cardiac echo was performed which was normal except for mild ventricular hypertrophy. Computed tomography aortogram revealed total occlusion of the abdominal aorta with collateral vessels formed by the left and right internal mammary artery (Figure 1-2).

A diagnosis of Takayasu arteritis was made based on the 1990 American College of Rheumatology (ARC) criteria. For control of blood pressure, in addition to amlodipine 10mg, valsartan 160mg and hydrochlorothiazide 12.5mg, the patient was started on atenolol 100mg. For immune suppression, we used 60mg prednisone followed by azathioprine 100mg as a steroid-sparing agent. The patient refused surgical treatment option.

The patient was managed with only oral medications and close outpatient follow-up. At one-year follow-up visit her blood pressure was 150/90, and she reported improvement of lower limb claudication. 

## Discussion

The ACR 1990 criteria for the classification of Takayasu arteritis include the age of disease onset ≤40 years, claudication of extremities, decreased brachial arterial pulse, blood pressure difference >10mm Hg between arms, bruit over subclavian artery or aorta and arteriography abnormalities. Three out of six criteria are to be met to make a diagnosis of Takayasu arteritis [3]. Our case fulfilled four out of the six above mentioned criteria.

Aizawa et al. reported a case of total occlusion of the abdominal aorta in a 30-year-old woman who was treated with oral medications for a period of over forty years. Middle-aged patients can have long-standing chronic inflammation which can lead to occlusion of the major vessels, but the chronic nature of the disease allows collateral vessels to be formed. Hence, such cases can be treated with oral medication alone without requiring vascular surgery [6]. In our case, the patient was only 21 years old and had massive collateral vessel formation by the left and right internal mammary artery. Another similar case was reported in a 35-year-old man who had complete occlusion of the abdominal aorta distal to the renal artery origin and had developed collateral vessels between the internal mammary and iliac arteries. The patient refused surgery and hence was treated with beta-blockers and angiotensin converting enzyme (ACE) inhibitors [7]. Kanda et al. described a case of Takayasu arteritis with complete occlusion of the thoraco-abdominal aorta in a 55-year-old man. A three-dimensional CT revealed collateral vessel formation with axillo-femoral bypass providing the predominant blood supply through the left internal iliac artery [8]. In yet another case, a 36-year-old woman had uncontrolled hypertension following an emergency Caesarian section at 29 weeks gestation because of high blood pressure of more than 180/100. She had complete obliteration of the abdominal aorta, bilateral renal artery stenosis, and collateral vessel formation. She was treated with prednisone and infliximab and at follow-up, after two months her blood pressure decreased to 130/70 [9].

The mainstay medical treatment of Takayasu arteritis is a steroid. However, treatment response to steroids is not always great. For instance, one study reported that only half of the patients on steroids respond to treatment [10]. Other steroid-sparing agents that have been studied include methotrexate, azathioprine, and cyclophosphamide. The best evidence supports the use of methotrexate as a steroid-sparing agent to which 50% of the patients respond. However, no single steroid-sparing therapy has proven to be more efficacious than the other. Therefore, side-effect profiles of these medications should be a deciding factor on which regimen to use [5]. 

The indication for surgical treatment is hypertension with renal artery stenosis, claudication, cerebral ischemia, aortic regurgitation and myocardial ischemia secondary to coronary artery involvement [11]. Wang et al. reported a case of Takayasu arteritis in a 44-year old man who presented with repeated attacks of heart failure, uncontrolled hypertension, and lower limb claudication. The imaging studies revealed occlusion of the abdominal aorta and bilateral renal artery stenosis. The patient underwent vascular bypass surgery. At one-year follow-up, the patient showed significant improvement in heart failure symptoms and better control of hypertension [12]. Our case illustrates that conservative medical therapy alone can be used to manage such patients due to the formation of collateral vessels. 

## Conclusions

Takayasu arteritis has a slow and chronic course. Occlusion of major vessels like abdominal aorta can progress slowly allowing for the formation of collateral vessels to bypass the obstruction. Hence, such cases can be treated conservatively with a close follow-up to monitor and prevent any long term complication. 
